# Design of Wideband Bandpass Filter Based on Corrugated Disk Resonator with Multiple Resonant Modes

**DOI:** 10.3390/ma14102614

**Published:** 2021-05-17

**Authors:** Qian Yang, Shuangyang Liu, Hongyu Shi, Kai-Da Xu, Xinyue Dai, Hao Du, Anxue Zhang

**Affiliations:** School of Information and Communications Engineering, Faculty of Electronic and Information Engineering, Xi’an Jiaotong University, Xi’an 710049, China; yangqiandianxin@mail.xjtu.edu.cn (Q.Y.); lsy6820@stu.xjtu.edu.cn (S.L.); hongyushi@mail.xjtu.edu.cn (H.S.); kaidaxu@ieee.org (K.-D.X.); daixinyue@stu.xjtu.edu.cn (X.D.); duhao1992@stu.xjtu.edu.cn (H.D.)

**Keywords:** bandpass filter, corrugated disk resonator, multiple resonant modes, spoof localized surface plasmon, transmission pole, wideband

## Abstract

A corrugated disk resonator with eight grooves is proposed for wideband bandpass filter (BPF) design. Due to the spoof localized surface plasmons resonances of the corrugated metallic structure, the dipole, quadrupole, hexapole modes, and a fundamental mode excited by the introduced short-circuited via holes are employed to realize four transmission poles (TPs) in the passband. The theoretical analysis is described by the electric field and current distributions on the resonator. The resonant frequencies can be tuned easily by the parameters of the structure, which can be used to adjust the center frequency and bandwidth of the BPF freely. Furthermore, two resonators are cascaded to obtain eight TPs to improve the selectivity performance. Finally, three fabricated filters demonstrate the design method.

## 1. Introduction

Metamaterials have been used for filter design for decades, such as in [1], where a resonant structure is characterized as an artificial magnetic conductor material which exhibits allowed and forbidden bands of mode propagation for designing a bandpass filter (BPF). A multilayer electromagnetically induced transparency (EIT) metamaterial structure is proposed for stopband filter design in [2]. The EIT metamaterial consists of a U-shaped resonator and a strip on a polyimide substrate which produce a coupling effect between multi EIT-like resonances to realize stopband performance. Another commonly used configuration is the artificial metamaterial transmission line, whose applications in filters design are investigated extensively [3,4,5]. Dual-composite right/left-handed transmission lines are used to realize bandstop performance [3] and single-, dual-, and tri-band bandpass characteristics [4,5] with compact circuit size.

On the other hand, surface plasmons can confine electromagnetic wave in a subwavelength scale to realize miniaturize components, which are also very useful in filters design. Surface plasmons are mainly divided into two categories by working principles. The propagating surface plasmon polaritons (SPPs) with waveguide configurations and the resonant localized surface plasmons (LSPs) with closed surfaces have been widely studied. Since metal has a negative permittivity in the optical frequency region, metallic particles display surface plasmon resonances which can be used to design optical components directly. However, it behaves akin to perfect electric conductors at lower frequencies, which do not support surface plasmons. Therefore, the plasmonic metamaterials with textured closed surfaces have been researched to support localized electromagnetic resonances at terahertz (THz) and microwave bands, which have similar properties to those of the spoof surface plasmon polaritions (SSPPs) or those designed with a highly confined surface electromagnetic wave [6,7,8,9,10,11,12,13,14,15,16,17,18,19,20,21,22,23,24]. SSPPs waveguide which works as lowpass filtering part integrated with coplanar waveguide, substrate waveguide, or bandpass filtering part are widely applied in wideband or dual-band BPFs design [10,11,12,13]. Surface plasmon resonances are usually applied in sensor, absorber, and frequency selective surface design [15,16,17,18,19]. In addition, due to the abundant resonant modes exciting a narrow frequency range [6,7,8,9], the spoof LSP integrated with substrate waveguide structure is proposed to design a wideband BPF with compact size [25] compared with the filters using SSPPs configuration.

There are many different analytical methods for investigating the spoof LSP resonators. The resonances of spoof LSPs in the textured cylinders were illustrated by a medium analytical approach in [6]. The characteristic mode analysis is applied in [15,21] to reveal resonant modes with eigencurrents distribution. A partially implicit finite-difference time-domain method is utilized for the wideband analysis of spoof LSPs in 2-D structures. The eigenmode method can be used to analyze the textured structure in microwave frequency band for resonances as well. The most used structures of spoof LSP resonator are the periodically corrugated disk structure [6,7,8,9,12,13,14,15,16,17,18] and the metallic spiral structure [22,23]. The resonant modes excited by a plane wave or a microstrip line in disk or ring resonators are depicted as dipole, quadrupole, hexapole, octupole modes, and so on. The sparse resonant modes can be employed to design multiband BPF [8] or wideband BPF [25]. However, the higher modes are difficult to excite, and the filters using higher modes have large insertion losses.

In this paper, a periodically corrugated disk with fewer grooves has been proposed, employing the dipole, quadrupole, and hexapole modes to design wideband BPF with good performance. Meanwhile, short-circuited via holes arranged in a circle at the center of the disk are introduced to excite a fundamental resonant mode, which is used to realize the four transmission poles (TPs) in the passband. Furthermore, two disks can be cascaded to produce eight TPs in the passband to improve the selectivity performance of the BPF. Compared with our earlier work in [26], the proposed filter and cascaded filter have more TPs in the passband with good selectivity performance. This paper is organized as follows. Section 2 shows the configuration of the designed resonator. The commercial simulation software HFSS is used to display the electric field distributions of the resonant modes for analysis in Section 3. Section 4 shows the discuss of the utilized resonances. The design approach is described in detail in Section 5. The simulated and measured results are shown in Section 6 for verification. Finally, a conclusion is given in Section 7.

## 2. Proposed Corrugated Disk Resonator Design

**Figure 1:**
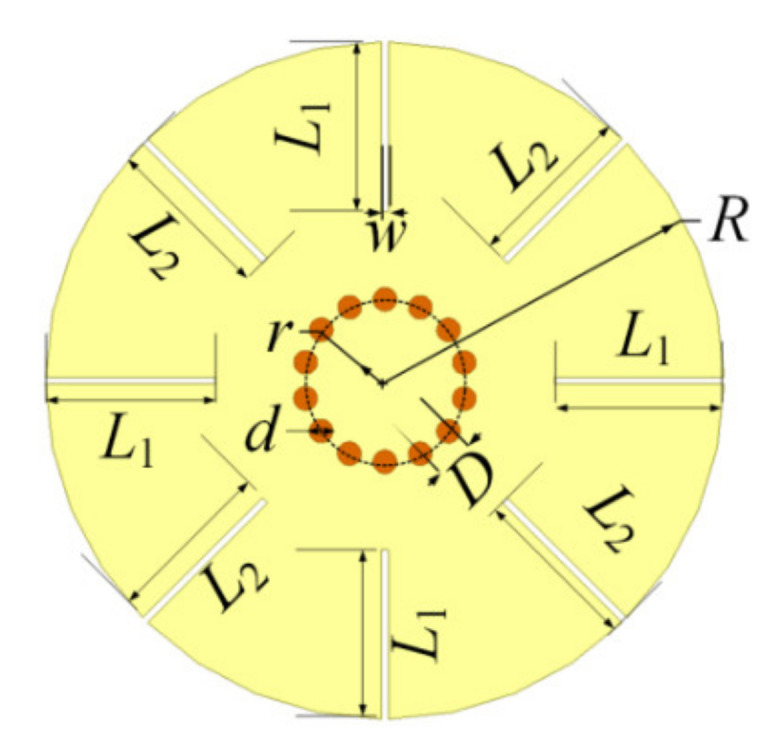
Structure of the proposed resonator.

The structure of the proposed resonator is shown in Figure 1. The resonator is printed on a substrate with relative dielectric constant of 2.65, thickness of 1 mm, and loss tangent 0.003. The upper metal surface of the substrate is etched with the textured configuration, while the bottom metal surface is remained as the grounded plane. It can be highly integrated with other printed circuit board fabricated components. The grounded via holes in Figure 1 are arranged in an inner circle with radius *r*, while the metallic disk has a radius *R*. As via holes play roles as an electric wall, so the parameters *d* and *D* should satisfy the conditions that *d* < 0.1*λg* and *D* < 4*d*, where *λg* is the wavelength in the substrate [27]. Four identical radial-oriented grooves with length *L*_1_ as well as another four grooves with length *L*_2_ are etched along the edge of the disk. For simplification, the widths of the eight grooves are set to be the same, denoted by *w*.

The resonator can be excited to produce resonances of spoof LSPs which are located adjacently by tuning the parameters of the resonator to be positioned in a passband for wideband BPF design. Eight grooves are used to excite less resonances compared with the configurations with much more grooves [6,7,8,9,20,21], which can be coupled to the input/output feeding lines properly with good performance.

## 3. Analysis of Resonances

**Figure 2:**
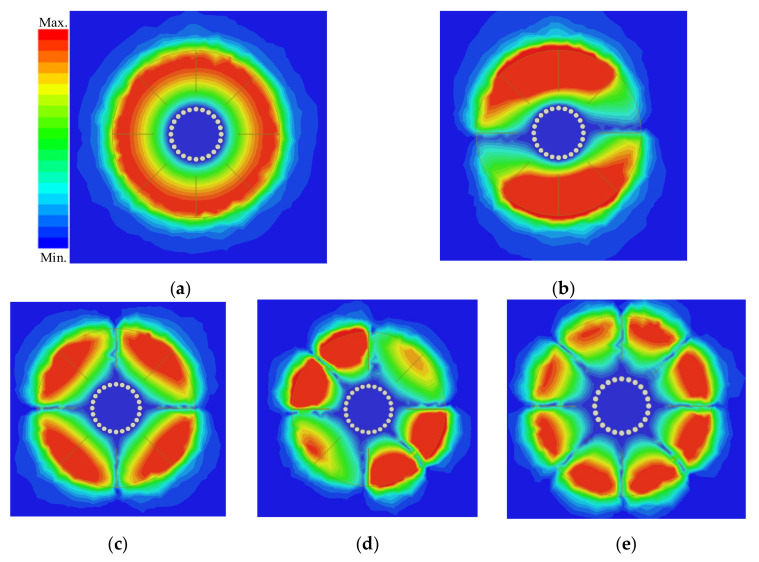
Electric field distributions of proposed resonator under different resonances. (**a**) M0. (**b**) M1. (**c**) M2. (**d**) M3. (**e**) M4.

The electric field distributions of the first five resonant modes of the proposed resonator in Figure 1 by eigenmode analysis are shown in Figure 2 with *r* = 3 mm, *L*_1_ = *L*_2_ = 5 mm, and *R* = 10 mm. Figure 2 gives the magnitude of the electric field, the value of which is positive. The resonant frequency of the fundamental mode M0 in Figure 2a is 4.7 GHz, while the other four resonant modes M1–M4 are located at 5.5, 7.0, 8.1, and 8.4 GHz, which are related to dipole, quadrupole, hexapole, and octupole modes, respectively.

## 4. Discuss

### 4.1. Discuss of Utilized Resonances

**Figure 3:**
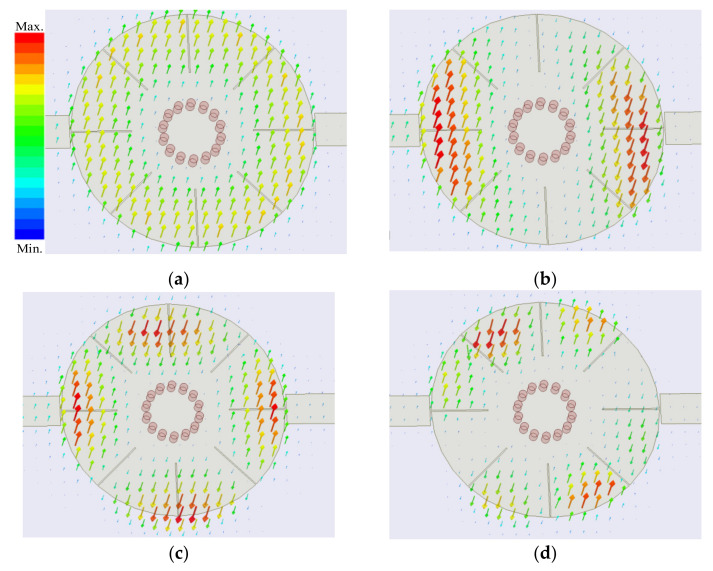
Electric field distributions of the first four resonant modes under weak coupling. (**a**) M0. (**b**) M1. (**c**) M2. (**d**) M3.

**Figure 4:**
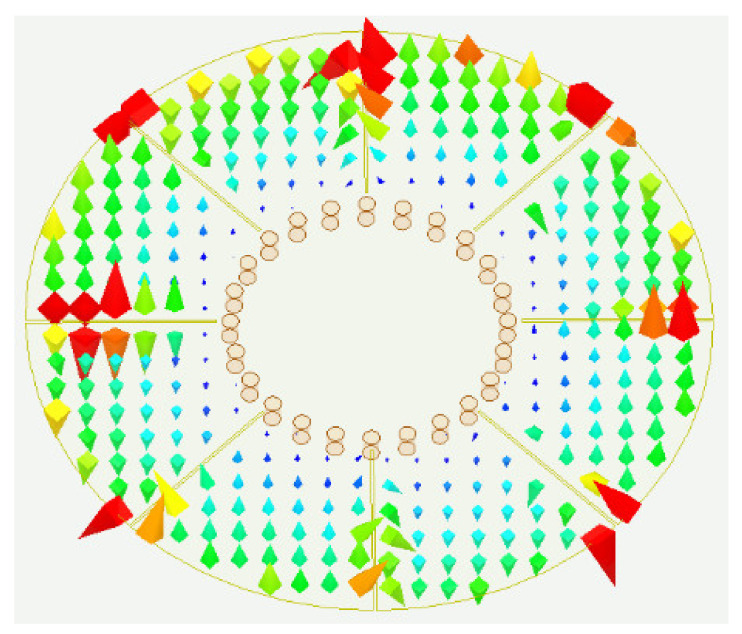
Electric field distribution of M4 mode on proposed resonator.

To study the useful resonances in wideband BPF design, the proposed resonator is excited by microstrip line. It is analyzed under weak capacitive coupling to investigate the electric field and surface current distributions of the excited resonant modes by the microstrip line, which are shown in Figure 3. As shown in Figure 3, the feeding lines excite resonances with the same electric field directions in the connecting region between the resonator and the feeding lines. Because each section of the resonator departed by the eight grooves has different electric field directions when the resonator works at the M4 mode, the connecting point has two opposite electric field directions, as shown in Figure 4. As a result, the M4 mode cannot be excited.

### 4.2. Discuss of Adjusting Resonances

**Figure 5:**
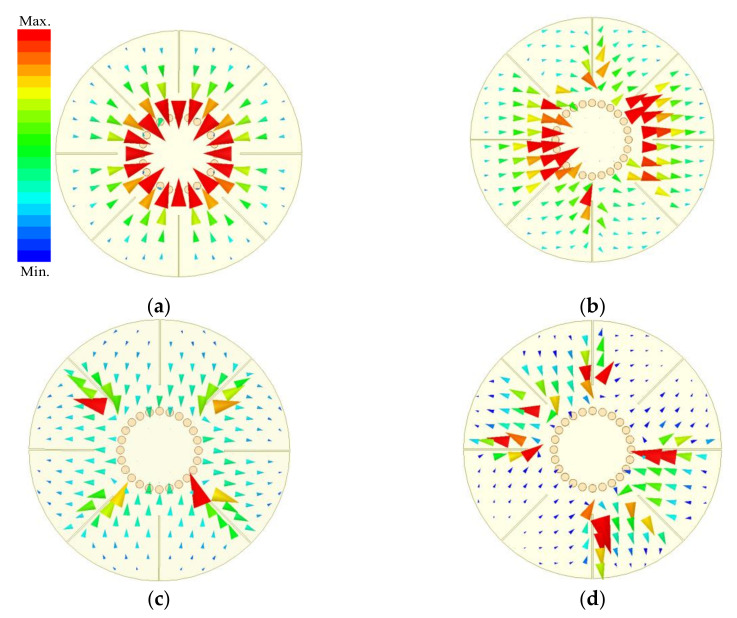
Current distributions of the first four resonant modes. (**a**) M0. (**b**) M1. (**c**) M2. (**d**) M3.

In order to utilize the multiple resonant modes excited by the short-circuited resonator to realize a wide passband, the frequencies of the resonances should be adjacent, and the resonant modes would be coupled appropriately to the feeding lines. So, the first four modes excited by the proposed filer configuration can be used to design a wideband BPF with a wide upper stopband. Meanwhile, the resonant frequencies should be tuned by the parameters of the structure to adjust the center frequency and bandwidth freely. The surface current distributions shown in Figure 5, related to the electric field distributions shown in Figure 3, can explain the effect of the grooves on the resonant frequencies, where the arrow direction indicates the current direction.

**Figure 6:**
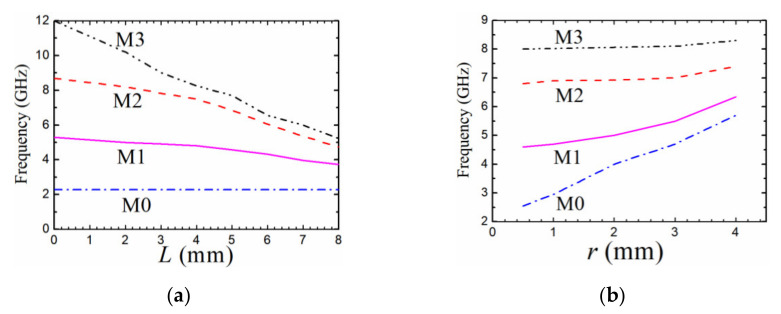
Variations of resonant frequencies with different parameters. (**a**) *R* = 10 mm, *r* = 0.5 mm, and *w* = 0.1 mm. (**b**) *R* = 10 mm, *L*_1_ = *L*_2_ = 5 mm, and *w* = 0.1 mm.

The grooves introduce major obstacles in the current paths of the M1, M2, and M3 modes, while they have no effect on the fundamental M0 mode as shown in Figure 5. Therefore, the grooves can bring down the resonant frequencies of the M1, M2, and M3 modes as shown in Figure 6a, where *L*_1_ = *L*_2_ = *L*. The longer the grooves are, the longer the currents of M1, M2, and M3 flow, which results in drawing down of the relevant frequencies. In addition, the radius of the inner circle *r* has great effect on the current distributions of M0 and M1 modes as shown in Figure 5a,b, which is also displayed in Figure 6b. The resonant frequencies of M0 and M1 modes grow larger when *r* increases while the resonant frequencies of M2 and M3 modes increase a little.

**Figure 7:**
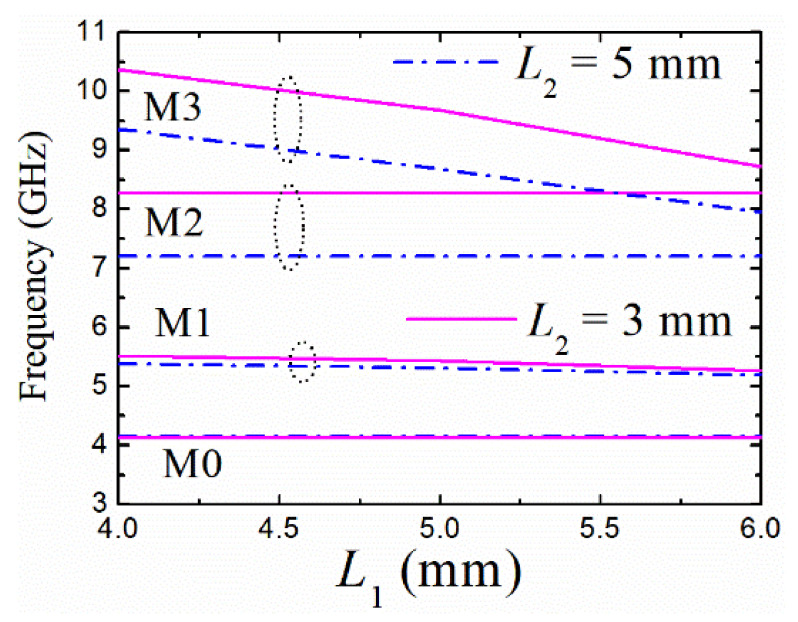
Variations of resonant frequencies with *L*_1_ and *L*_2_ (*R* = 10 mm, *r* = 2.4 mm, and *w* = 0.1 mm).

Since the current distributions shown in Figure 5 are excited by the feeding lines parallel to the slots, the M2 mode can be only tuned by the four grooves with length *L*_2_ as shown in Figure 5c, while the M3 mode can be tuned by all the eight grooves. That is to say, the resonant frequency of the M3 mode can be independently adjusted by the four slots of length *L*_1_ which have no effect on the resonant frequency of the M2 mode. The observed results can also be demonstrated by the variation curves of the resonant frequencies with different groove lengths plotted in Figure 7. Therefore, the resonant frequencies of M1, M2, and M3 modes can be brought down to close to that of M0 mode with the increasing lengths of the grooves. As a result, the bandwidth of the BPF based on the first quad-mode can be tuned easily by changing the lengths of the grooves.

## 5. BPF Design

### 5.1. Design of Four-Pole BPF

Based on the discussion in Section 4, the frequencies of the first four resonant modes have bigger intervals when the parameter *r*/*R* is smaller, which can obtain an ever-wider passband bandwidth. As shown in Figure 6a, when the inner circle is reduced to a pin, the resonator can be used to design BPFs with a much wider bandwidth. In this case, the minimum bandwidth can be realized when the grooves have the longest length. As shown in Figure 6a, the resonant frequencies get close when the length of the slots *L* gets larger, it results in a minimum 3-dB FBW. The bandwidth can be approximately deduced from the frequencies of the first and fourth resonant modes *f_M_*_0_ and *f_M_*_3_, FBW = 2(*f_M3_* – *f_M_*_0_)/(*f_M_*_0_ + *f_M_*_3_). It can be calculated from Figure 6a that the minimum value is about 78% when *L* = 8 mm. Therefore, when designing a BPF with a bandwidth larger than 78%, the resonator with a shorted pin should be chosen.

On the contrary, the value of *r*/*R* becomes larger, the first quad-mode get closer as shown in Figure 6b, which results in a less wide bandwidth. The narrower the bandwidth, the larger the ratio *r*/*R* should be selected. It can be calculated by the resonant frequencies of the first quad-mode in Figure 6b that the minimum 3-dB fractional bandwidth (FBW) is nearly 30% when *r* = 4 mm. Therefore, when designing a BPF with a 3-dB FBW between 30% and 78%, the resonator with a proper value of *r*/*R* should be selected. Since the first resonant mode M0 cannot be tuned by the slots, the frequency of the M0 mode can be used to determine the lower sideband frequency of the passband.

After the analysis of the parameters of the resonator, the design guidelines of the wideband BPFs can be summarized as follows.

(1) Determine the required specifications of BPF, the bandwidth, the center frequency, and the lower sideband frequency, which approximates to the resonant frequency of the M0 mode.
(2) According to the bandwidth, choose a value of *r*/*R*, the lower sideband frequency of the passband and the value of *r*/*R* can be used to determine the parameters *r* and *R*.
(3) The lengths of the grooves can be determined by the bandwidth.
(4) Finally, the length *L*_1_ and *L*_2_ can be slightly tuned to have good return losses in the passband.

### 5.2. Design of Eight-Pole BPF

**Figure 8:**
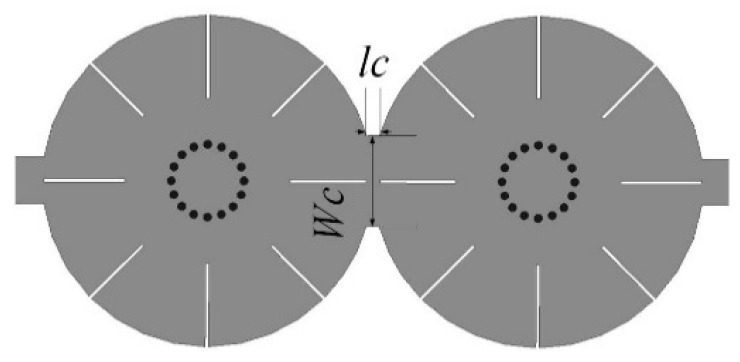
Two cascaded quad-mode resonators.

Two quad-mode resonators can be cascaded to improve the selectivity and realize eight-order BPF design. The structure is shown in Figure 8 using two resonators connecting directly for proper coupling. The coupling values between the two resonators can be tuned by the width and length of the connecting part. The center frequency and bandwidth can be determined by the analysis in Section 5.1.

## 6. Simulations and Measured Results

**Figure 9:**
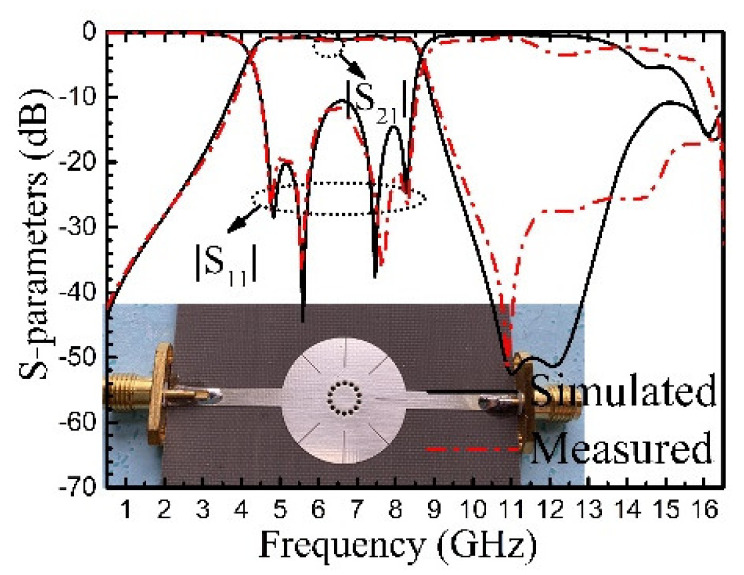
Measured and simulated responses of BPF-I with photograph inset.

Three filters have been designed for the verification of the theoretical analysis in Section 5. The first filter (BPF-I) has a passband from 4.21 to 8.75 GHz with center frequency (*f*_0_) of 6.5 GHz and a 3-dB FBW of 70%. The final dimensions are optimized by the full-wave electromagnetic simulation software ANSYS, which are *R* = 10 mm, *r* = 2.4 mm, *L*_1_ = 5 mm, *L*_2_ = 5 mm, and *w* = 0.1 mm. The 50 Ω feeding lines are directly connected to the resonator. The fabricated filter is shown in Figure 9 with frequency responses. The filter size is 0.65*λg* × 0.65*λg*, where *λg* is the guided-wavelength at the center frequency. The results not only demonstrate the analysis in Section 4, but also show good agreement between simulation and measurement in the working frequency range. The measured insertion loss (IL) at center frequency is 1.6 dB, which is caused by the radiation, dielectric, and conductor loss. The measured return losses are larger than 10.2 dB in the passband. The stopband with 20 dB suppression can be extended to 2.3 *f*_0_.

**Figure 10:**
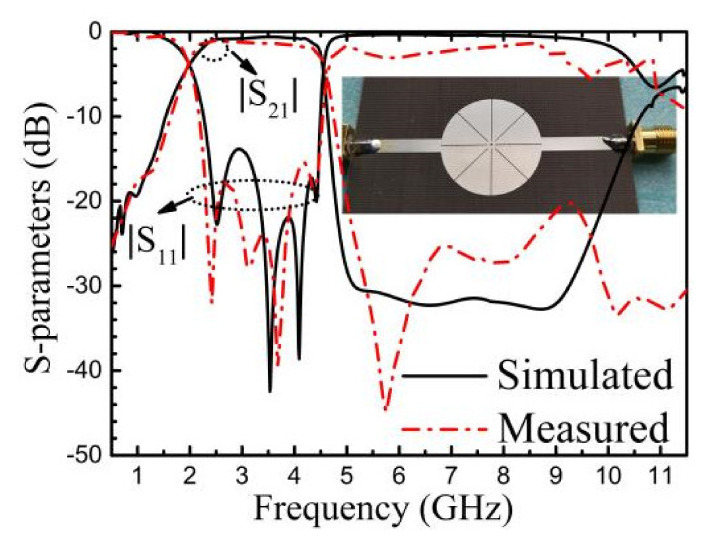
Simulated and measured results of BPF-II.

The second filter (BPF-II) is designed to operate at a much lower center frequency of 3.3 GHz with the same size as that of BPF-I. The passband is from 1.97 to 4.56 GHz with a 3-dB FBW of 79.3%. In order to reduce the filter size, the radius of the inner circle is decreased to be zero to have a minimum fundamental resonant frequency of the M0 mode, and the lengths of the slots are chosen to be the maximum value at the same time to lower down the other three resonant frequencies. The optimized parameters are *R* = 10 mm, *L*_1_ = 9.2 mm, *L*_2_ = 8.9 mm, and *w* = 0.1 mm. The fabricated filter with frequency responses are shown in Figure 10. The filter size is 0.33 *λg* × 0.33 *λg*. The upper stopband is from 5 to 10 GHz with insertion loss larger than 20 dB, which means that the spurious harmonic suppression extends to 3*f*_0_. The measured insertion loss at the center frequency is 1.4 dB. The measured return losses in the passband are larger than 15 dB. The discrepancy between the simulated and measured results are mainly due to the fabrication tolerance, the insertion losses, and radiation losses of the sub miniature version A (SMA) connectors, and inaccurate permittivity of the substrate.

**Figure 11:**
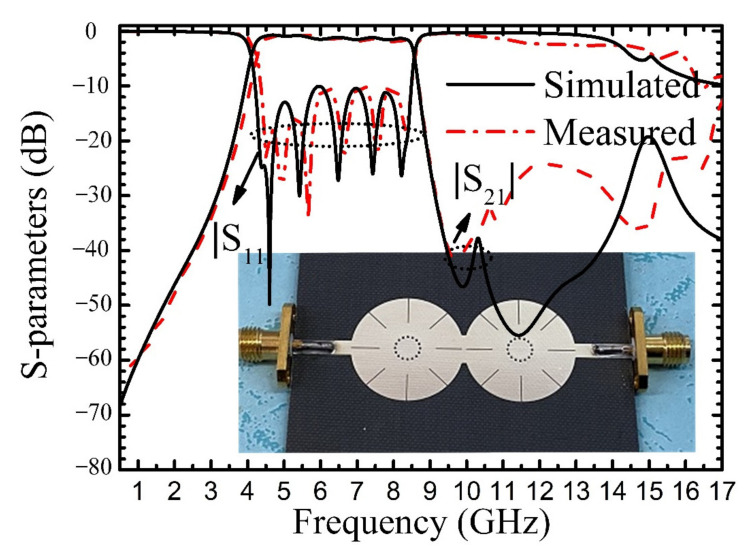
Simulated and measured results of BPF-III.

The simulated and measured responses with fabricated filter using two quad-mode resonators are shown in Figure 11 with parameters of *R* = 10, *r* = 2.2, *L*_1_ = 5, *L*_2_ = 4.5, *w* = 0.1, *Wc* = 5.5, and *lc* = 0.8 (units in mm). Since the resonant frequencies of one resonator are very close to those of the other, it cannot be seen eight-pole in the passband. The measured center frequency is 6.43 GHz and FBW is about 66.3%. The maximum insertion loss is 1.7 dB, and the minimum return loss is 10 dB in the passband. The measured upper stopband suppression larger than 20 dB can extend to 2.6*f*_0_. Table 1 shows a comparison of the proposed filters with BPFs based on the multiple resonant modes. It can be seen that the quad-mode used in the proposed short-circuited circular patch resonator can realize wide passband bandwidth and the higher spurious resonant modes can be suppressed by the feeding structure.

## 7. Conclusions

This paper has presented a novel short-circuited corrugated disk resonator for wideband BPF design. The excited fundamental mode is M0 mode. The frequency of the M0 mode is much lower than that of the second resonant mode M1. Meanwhile, due to the tapped feeding lines along the slot line, the first quad-mode can be employed to form a wide passband with a wide upper stopband. The bandwidth and center frequency can be easily tuned by the length of the grooves and the radius of the inner circle in a wide range. Two cascaded resonators can be used to improve the performances of the wideband BPF design. The experimental results show good agreement with the theoretical ones, which demonstrates the design principle.

## Figures and Tables

**Table 1:** Comparison with related references.

Ref.	*f*_0_	IL (dB)	FBW (%)	Modes	Stopband	Size (*λg* × *λg* )
[10]	8.5	1.5	35.3	--	1.5*f*_0_	4.85 × 0.92
[12]	65	2.0	50.5	3	--	0.86 × 0.16
[24]	10.2	0.8	63.0	8	--	1.28 × 1.28
BPF-I	6.5	1.6	70.0	4	2.3*f*_0_	0.65 × 0.65
BPF-II	3.3	1.4	79.3	4	3*f*_0_	0.33 × 0.33
BPF-III	6.4	1.7	66.3	8	*2.6* *f*_0_	1.28 × 0.63

*λg*: Guide wavelength on the substrate at the center frequency.; *f*_0_: Center frequency of the BPF, unit in GHz.

## Data Availability

The data presented in this study are available on request from the corresponding author.
